# Corneal Epithelial Defects in Diabetic Patients Following Pars Plana Vitrectomy

**DOI:** 10.1155/joph/8873950

**Published:** 2025-04-24

**Authors:** Kristin Ates Hicks, Yujia Zhou, Jay Talati, Khushi Saigal, Joshua Kalish, Shivani Shah, Siva Iyer, Lauren Jeang

**Affiliations:** ^1^Department of Ophthalmology, University of Florida, Gainesville, Florida, USA; ^2^College of Medicine, University of Florida, Gainesville, Florida, USA; ^3^Vitreoretinal Associates, Gainesville, Florida, USA

**Keywords:** corneal epithelial defect, diabetes mellitus, pars plana vitrectomy

## Abstract

Diabetes mellitus is a known risk factor for corneal epithelial defects (CEDs) after pars plana vitrectomy (PPV), but it is unclear if diabetes severity or specific diabetic risk factors are associated with an increased risk of CED. The purpose of this retrospective cohort study was to identify factors associated with CED and healing time in association with diabetes severity in diabetic patients following PPV. The electronic health record database at University of Florida in Gainesville was queried to identify patients who underwent PPV for retinal detachment (RD) between April 2016 and April 2022. Patient charts were reviewed for clinical data including type of diabetes (if present), diabetes duration and severity, and associated diabetic comorbidities. The main outcome measures included presence of a CED within one month postoperatively, treatment of CED if present, and CED healing time. A total of 637 patients were analyzed, with a total of 243 eyes (26.5%) that belonged to diabetic patients. The diabetic patients were further separated into a proliferative diabetic retinopathy (PDR) group and a nonproliferative diabetic retinopathy (NPDR) group. Diabetes was associated with the development of an initial CED (*p*=0.040), consistent with existing literature. There was not a significant difference in CED risk when comparing NPDR and PDR patients, although PDR patients tended to have more severe long-term outcomes with persistent corneal epithelial defects (PCEDs). This suggests that PDR patients may still require closer monitoring and earlier intervention for postoperative CED following PPV, as compared to the NPDR patient population.

## 1. Introduction

Corneal epithelial defects (CEDs) are areas of disrupted epithelium on the cornea surface, often caused by trauma or ocular disease. Under normal conditions, the epithelial layer heals within 7–14 days after undergoing a complex active repair process [1–3]. If the CED fails to heal within this time frame, despite standard supportive treatment, it is considered a persistent corneal epithelial defect (PCED), in that the CED has not healed after 14 days [2]. CEDs are often painful and can disrupt vision, and the risks of significant complications such as infection and vision loss increase with PCEDs and recurrent corneal erosions (RCEs) [2, 4]. In severe cases of prolonged epithelial healing, visually significant scar formation may occur, requiring corneal transplantation [5].

Often, CEDs appear after pars plana vitrectomy (PPV) [6, 7] and multiple studies have associated this with diabetes, older age, and longer surgical times [6–13]. The reason for this association between diabetes, CED, and PPV is unclear, but there is evidence that corneal innervation is altered in diabetic patients and after vitrectomy [14, 15]. Diabetics could be prone to neurogenic tear dysfunction and chronic ocular surface inflammation leading to poor epithelium maintenance [16]. Changes in corneal metabolism from diabetes can cause endothelial cell damage and stromal thickening [17], which in turn may lead to weakened epithelium and slower postoperative visual recovery.

The severity of diabetes (i.e., poor blood glucose control) is associated with the severity of diabetic retinopathy [18]. However, there is limited information in the current literature on whether there is an increased risk of CED, PCED, and longer healing time postoperatively with more severe diabetic disease. We hypothesized that the severity of diabetes may also be associated with severity of postoperative corneal complications and delayed healing. Additionally, microvascular complications of diabetes such as diabetic nephropathy and peripheral neuropathy are well described, but it is not known if these microvascular complications correlate with a higher risk of CEDs after PPV. Therefore, this study aims to examine whether diabetes severity, classified as nonproliferative diabetic retinopathy (NPDR) or proliferative diabetic retinopathy (PDR), is associated with increased risk of CED and PCEDs.

## 2. Materials and Methods

### 2.1. Subjects and Study Design

This is a retrospective cohort study in which the electronic health record database at University of Florida in Gainesville was queried to identify diabetic patients who underwent PPV for retinal detachment (RD) between April 2016 and April 2022. CPT codes used in the chart review included 67113 and 67108. Patients over 18 who underwent PPV for RD were included. Patients who underwent PPV for any other reason (i.e., epiretinal membrane, macular hole, dislocated lens, lens exchange, globe trauma, and retained lens fragments), patients who had scleral buckle placement without PPV for RD, and reoperations (i.e., second PPV/intraocular surgery) were excluded. The study was approved by the Institutional Review Board of University of Florida (IRB202102383) and was conducted in accordance with the Declaration of Helsinki.

Data points recorded during chart review included patient age, the left or right operative eye, prior ocular history, prior ocular surgical history, diabetes diagnosis, diabetic retinopathy stage (NPDR or PDR), recent preoperative hemoglobin A1C (HbA1c), maximum recorded HbA1c, duration of diabetic disease, and presence of diabetic neuropathy and/or nephropathy. Intraoperative measures included gauge size used in PPV, use of gas tamponade or silicone oil, use of cryotherapy, use of laser, surgical length time (in minutes), use of a viscoelastic device on the cornea, and use of corneal scraping. Outcome measures included incidence of CEDs identified within 1 month after PPV, duration of CED in days from diagnosis to resolution, and incidence of PCEDs defined as CED with a healing time longer than 14 days. We acknowledge that due to the nature of this retrospective study with the above exclusion criteria, this may include unavoidable selection bias. We have attempted to limit this as much as objectively possible.

### 2.2. Statistical Analysis

JASP statistical software Version 0.19.0 with *α* = 0.05 was used for all statistical analyses (The JASP Team, Amsterdam, The Netherlands). Welch's *t*-test was used to compare age, HbA1c, and healing time between PDR and NPDR groups. Welch's *t*-test was also repeated dividing patients into groups by neuropathy and nephropathy. Fisher's exact test was used to compare rates of CED outcomes by diabetes or PDR and NPDR groups. The Mann–Whitney *U*-test was used to compare surgery length for those with and without CEDs. Pearson correlation was used to estimate any association between healing time and age or HbA1c measurements. Multivariate logistic analysis was considered but due to low incidence < 50% and numerous variables of interest, multivariate regression would not be possible.

## 3. Results

### 3.1. Baseline Characteristics

A total of 637 eyes were included in this retrospective study. The mean age at time of PPV was 57.3 years (SD 13.8). 243 eyes (38.1%) belonged to diabetic patients, with 76 in the NPDR group and 167 in the PDR group. 93 eyes (14.6%) belonged to patients with concurrent nephropathy while 125 eyes (19.6%) belonged to patients with concurrent peripheral neuropathy. 394 eyes (61.8%) belonged to nondiabetics, which served as the control group. The mean recent preoperative HbA1c was 8.2 (SD 2.13) while the mean maximum recorded HbA1c was 9.8 (SD 2.754).

**Figure 1:**
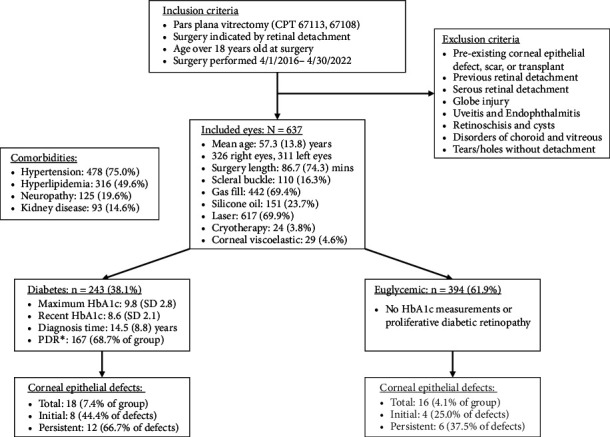
Patient flowchart. The diagram shows inclusion and exclusion criteria used for screening the electronic medical record. Selected comorbidity rates are shown for included patients, and details of the patient's eye surgery are described. Groups of diabetic and nondiabetic patients were evaluated after vitrectomy with corneal epithelial defect outcomes listed in the lowest boxes. ^∗^Proliferative diabetic retinopathy.

Among all included eyes, 34 (5.3%) developed CED within 30 days after PPV, with 18 eyes belonging to diabetics and 16 eyes belonging to nondiabetics. Of those 34 eyes, 18 eyes took longer than 14 days for CED resolution and were diagnosed as PCED. Other patient and surgical characteristics are listed in Figure 1.

### 3.2. Characteristics of PDR and NPDR Patients

**Figure 2:**
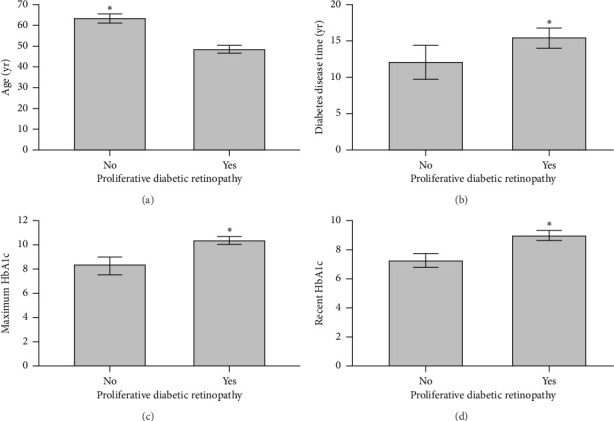
Characteristics of patients with proliferative diabetic retinopathy. Bar charts with a 95% confidence interval are displayed comparing the (a) age of patients, (b) duration of diabetic disease, (c) maximum HbA1c, and (d) maximum HbA1c for patients with no proliferative diabetic retinopathy and for patients with proliferative diabetic retinopathy. ^∗^indicates significance of *p* > 0.05 by Welch's *t*-test.

When comparing patient characteristics in diabetic patients with PDR and those with NPDR, we found that patients with PDR were younger with a mean difference of 15 years (*p* < 0.001), had a higher preoperative HbA1c and maximum HbA1c (*p* < 0.001), and had longer diabetes disease duration (*p*=0.016). Characteristics are outlined in Figure 2.

### 3.3. Risk Factors for CED

**Figure 3:**
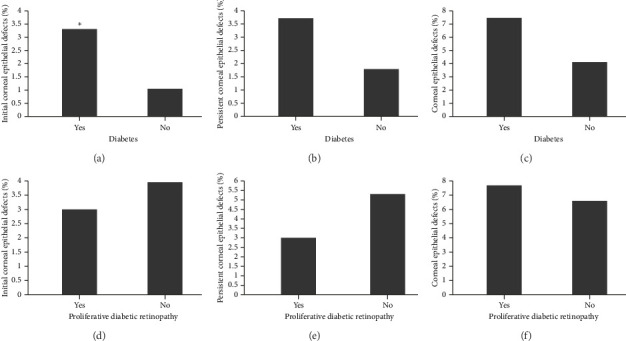
Rates of corneal epithelial defects. Bar charts compare the rates of (a) initial corneal epithelial defects, (b) persistent corneal epithelial defects, and (c) total corneal epithelial defects of patients with and without diabetes after pars plana vitrectomy. Similar bar charts compare the rates of (d) initial corneal epithelial defects, (e) persistent corneal epithelial defects, and (f) total corneal epithelial defects when comparing diabetic patients with and without proliferative diabetic retinopathy. ^∗^indicates *p* > 0.05 by Fisher's exact test.

**Figure 4:**
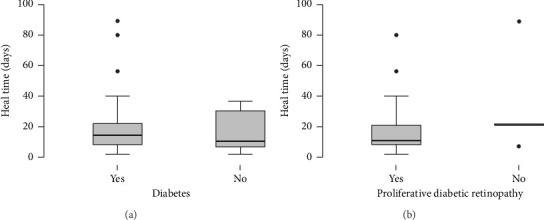
Healing time for corneal epithelial defects. Box plots compare the corneal healing time for (a) diabetics compared to nondiabetics (*p*=0.255) and for (b) diabetic patients with proliferative diabetic retinopathy compared to those without (*p*=0.530). Outliers are shown with dots.

The diagnosis of diabetes is associated with the development of an initial CED (defined as the presence of an epithelial defect on postoperative day (POD) 0 or 1) (Figure 3(a), **p**=0.040). Furthermore, the diagnosis of diabetes approaches statistical significance in association with the development of a CED within 30 days postoperatively (Figure 3(b), **p**=0.068). The presence of PCED among CEDs approaches statistical significance in diabetics, although it is a weaker correlation (Figure 3(c), **p**=0.089). There was no association in the prevalence of initial CEDs, total CEDs, or healing time/PCEDs when comparing NPDR diabetics and PDR diabetics (Figures 3(d), 3(e), and 3(f)). We did not see a significant difference in the rate of CED healing time when comparing the control group to diabetics and when comparing NPDR to PDR patients (Figure 4). There were no associations between development of CEDs and patients with diabetic nephropathy and/or neuropathy.

**Figure 5:**
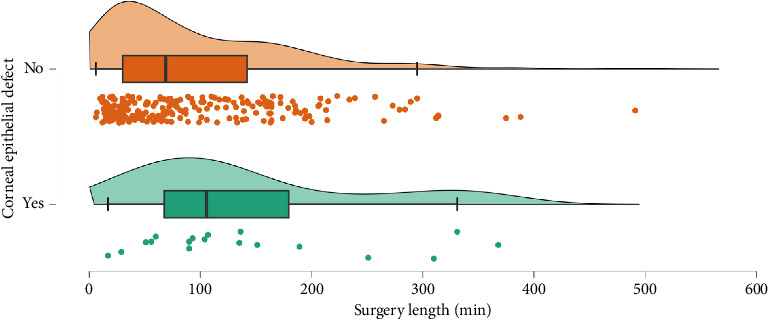
Raincloud chart of surgery length. Box plots comparing surgery length in patients with (green) and without (orange) corneal epithelial defects after vitrectomy show that longer surgery length was significantly associated with corneal epithelial defects after pars plana vitrectomy by Mann–Whitney *U*-test (*p*=0.021) with a small effect size. Interpolated distribution curves are shown above the boxes and individual points are plotted below.

Surgical length appears to be the best intraoperative predictor for risk of CEDs (Figure 5, **p**=0.021). Eyes with CEDs had a median intraoperative time of 97.5 min while eyes without CEDs had a median intraoperative time of 61.0 min. When analyzing intraoperative risk factors such as vitrectomy gauge, use of gas or silicone oil, cryotherapy, laser, scleral buckle, and use of a viscoelastic device on the cornea, we found no significant difference or trend regarding incidence of CEDs. Corneal scraping was not performed on any of the patients that met inclusion criteria.

## 4. Discussion

Our retrospective study of 637 patients over a 6-year span investigating CEDs following vitrectomy for RD shows that the diagnosis of diabetes was significantly associated with the development of an initial CED. This finding is consistent with the literature, in which multiple studies have identified diabetes mellitus as a risk factor for development of postoperative CED after PPV [7–11]. To the best of our knowledge, our study is the largest retrospective study that encompasses numerous preoperative and intraoperative factors in data analyses, rendering strong internal validity. This unique investigation showed no significant association between diabetes severity and incidence of epithelial defects. However, when an epithelial defect did occur in a patient with PDR, the outcomes were worse.

**Figure 6:**
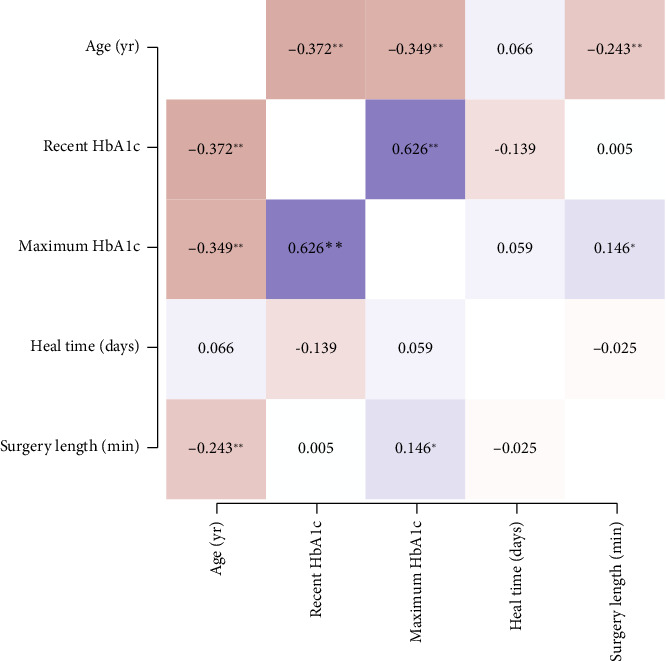
Correlation matrices of HbA1c with age, surgery length, and healing time. Red intensity indicates degree of a negative correlation, and purple intensity indicates degree of a positive correlation. ^∗∗^indicates *p* < 0.001 by Pearson correlation and ^∗^indicates *p* < 0.05 by Pearson correlation.

This is the first study to examine distinctions among diabetics, including retinopathy status, HbA1c, and diabetic disease duration as it relates to CED after PPV. The rates of immediate postoperative CED and overall postoperative CED were not significantly associated with PDR. Furthermore, we did not find a difference in CED healing time between nondiabetics and diabetics nor between NPDR and PDR patients. We attempted to stratify NPDR into the different stages (e.g. mild, moderate, and severe NPDR) but found that they were not statistically different from each other in our dataset. We also attempted to stratify by diabetes disease duration, but this was limited by poor documentation of disease duration. Our PDR patient cohort tended to be younger, have higher preoperative and maximum lifetime HbA1c, and longer diabetic disease duration. A correlation matrix consisting of HbA1c with age, CED healing time, and surgical length was performed to further investigate the data (Figure 6). We found that recent HbA1c and maximum HbA1c were highly correlated. This correlation may simply be the result of having only one documented HbA1c or due to the recent HbA1c being the maximum recorded. Additionally, older age was significantly correlated with lower HbA1c. Healing time was not significantly correlated with age, HbA1c, or surgery length (Figure 6). We propose that age may be serving as a confounding factor in CED healing time. Although our younger patients tended to have poorer glucose control, their rate of healing may be similar to our older patients who have impaired healing due to aging. Aging has been associated with a decrease in corneal nerve density, decrease in corneal sensation, and age-related changes to the ocular surface such that older patients have poorer epithelial and endothelial cell integrity [12, 19, 20]. Further examination of diabetes severity with better age matching is needed to discern the impact of both diabetes and age on epithelium wound healing.

In our analysis, we found that longer surgical time was the only significant intraoperative predictive factor for development of CEDs, which is also consistent with the literature [6, 8, 10, 11, 13]. Although there was no statistically significant difference in CED and PCED incidence, we found that PDR patients tended to have more severe long-term outcomes with postoperative CEDs, requiring multiple interventions with bandage contact lenses (BCLs), temporary tarsorrhaphy, and eventual penetrating keratoplasty (PKP) for corneal opacification. This may be related to factors such as increased epithelial fragility and altered epithelial basement membrane composition in severe diabetes and diabetic retinopathy compared to nondiabetics and diabetes without retinopathy [21–23]. In addition, longer duration of hyperglycemic exposure to the cornea causes impairment of corneal and conjunctival epithelial cell barrier functions with alterations in the molecular pathways involved in normal epithelial wound healing [24, 25]. This is clinically significant in that it suggests PDR patients may benefit from earlier postoperative interventions and closer postoperative follow-up, as discussed later in this section.

Only 1 out of the 16 CEDs among nondiabetics was documented to have long-term corneal scarring, with a heal time of 14 days without requiring invasive procedures. In contrast, 5 out of the 18 CEDs among diabetics required temporary tarsorrhaphy due to nonhealing epithelial defects, with 4 out of the 5 patients having high-risk PDR (Table 1). For Patient 1 and Patient 3, PKP was performed for corneal thinning and persistent stromal edema with scarring, respectively. Severe concurrent corneal and retinal disease often resulted in patients reaching a monocular status at a young age. Given the potentially devastating sequelae, thorough postoperative assessment of the corneal epithelium in younger diabetic patients with PDR may be beneficial. Encouragingly, the best outcome was in one of the younger patients (Table 1, Patient 2) in which the CED was identified and managed early. This suggests that earlier proactive intervention can lead to better outcomes for corneal health in this population. This includes earlier interventions with more frequent use of lubricating artificial tears, artificial tear ointment, punctal plugs, BCLs, use of amniotic membranes, and/or temporary tarsorrhaphy. Recently, the use of topical insulin has been shown to significantly improve the rate of epithelial healing in eyes with neurotrophic keratopathy after diabetic vitrectomy [26]. Thus, one can consider initiation of topical insulin early in the postoperative period specifically for PDR patients with a CED. Additionally, one can consider referral to a cornea specialist for comanagement if there are any signs of prolonged healing in this population.

We hypothesized that presence of diabetic nephropathy and neuropathy would be associated with increased risk of CEDs and/or PCEDs owing to the fact that diabetic microvascular diseases are associated with severity and duration of disease [27–29], but no statistical significance was found. This may be due to variation in documentation of nephropathy and neuropathy by providers. In our review of the data, we primarily looked at diagnostic codes for nephropathy and neuropathy, but it is possible that not all primary care providers included this in the patient diagnoses. Additionally, some patients may have had early signs of diabetic neuropathy, but objective measurements were not obtained such as detailed sensory testing, reflex testing, and overall muscle strength and tone. Similarly, early detection of diabetic nephropathy may be limited by lack of routine testing for urine albumin. Perhaps additional prospective studies may help elucidate this association in which more objective physical examination measurements are obtained in the diabetic and nondiabetic patient cohorts.

Compared to other studies, we found a lower percentage of CEDs, which is likely due to our more stringent definition of CED and may also be reflective of our cohort population [7–9, 13]. Unlike Chen et al. [7] and Hiraoka et al. [13], we did not include presence of punctate epithelial erosions, superficial punctate keratopathy, or corneal edema in our definition of a clinically significant CED. This difference in inclusion criteria likely accounts for the lower percentage of CEDs recorded in our study compared to other studies. Furthermore, Sarici et al. [10] had a similar sample size of 856 eyes with a total of 94 CEDs in a cohort of 856 eyes. They also found that surgery length was significantly associated with increased incidence of CEDs with a mean time of 100 ± 67 min. This is similar to the median surgical time among our CED patients. Perhaps differences in surgeon's use of intraoperative lubricating agents on the cornea, which is often not recorded in the operative reports, may be influential for the difference in incidence of CEDs. Similarly, differences in surgical approach with differences in intraoperative perfusion pressures and frequency of scleral compression may account for difference in incidence of CEDs. This would benefit from a prospective (or retrospective) study, in which intraoperative factors are analyzed or recorded more closely, such as frequency of administration of intraoperative lubricating agents, perfusion pressures during vitrectomy, and frequency of scleral compression intraoperatively.

Our study was primarily limited by postoperative follow-up and generalizability. Our study includes one of the largest and most inclusive cohorts on this topic from a single institution. However, patients were typically only seen on POD0/1, POD7, and POM1. Thus, the duration of documented CEDs is not precisely recorded (e.g. some CEDs likely healed before the patient's follow-up appointment). Due to the retrospective nature of the study, it is difficult to accurately collect and analyze the specific postoperative treatments applied to each patient. To circumvent that, it would require prospective daily monitoring of postoperative patients, which is not truly feasible in a busy academic or private practice setting. Likewise, Sarici et al. [10] and Chiang et al. [8] also state that patients were typically seen at POD0/1 and then POW1, and thus there may be an overestimation of the CED heal time since the eyes were not followed daily. Combining data from multiple centers with different follow-up schedules may resolve this shortcoming and improve the generalizability of this study for diabetic patients in other locales. Suggested future directions include exploring the risk of CEDs/PCEDs associated with diabetes severity with a larger sample size and perhaps more frequent monitoring in the postoperative period. A more detailed investigation of CED and its association with the presence of diabetic nephropathy and neuropathy may also lend more information regarding risk factors for retinal surgeons in preoperative planning.

Our study is the first of its kind to explore diabetes severity and its correlated risk of postoperative CEDs following PPV. We found that diabetes is associated with increased risk for initial CED after PPV. We found no association between CED outcomes and PDR, but patients with PDR tended to have more persistent and severe complications following postoperative CEDs. Thus, not only the presence of diabetes but also the severity of diabetic retinopathy should be considered in preoperative planning and postoperative management.

## 5. Conclusion

This retrospective cohort study confirms that diabetes is associated with increased risk for initial CED in patients undergoing PPV for RD repair. Furthermore, although there was no significant difference when comparing risk of CEDs with that of diabetic severity, when a CED does occur, PDR diabetic patients still tended to have worse, long-term complications affecting their vision.

## Figures and Tables

**Table 1:** Diabetic patients requiring advanced treatment interventions for postoperative CEDs.

Patient #	Age (years)	Diabetic retinopathy severity	CED onset (post-op day)	Healing time (days)	Interventions	Outcome/final postoperative vision (VA)
1	59	Mild	22	22	Tarsorrhaphy, PKP	Clear transplant VA: 20/400
2	31	High-risk PDR	4	2	Tarsorrhaphy	Healthy VA: LP
3	28	High-risk PDR	22	^∗∗^	Tarsorrhaphy, amniotic membrane, keratoprosthesis, PKP	Transplant failure VA: LP
4	57	High-risk PDR	28	34	BCL, tarsorrhaphy	Central scar VA: 20/200
5	34	High-risk PDR	1	5	Tarsorrhaphy	Recurrent epithelial erosions, central scar VA: LP

Abbreviations: BCL, bandage contact lens; LP, Light perception; PKP, penetrating keratoplasty.

^∗∗^Did not heal, eventually required PKP.

## Data Availability

The data that support the findings of this study are available on request from the corresponding author. The data are not publicly available due to privacy or ethical restrictions.

## Nomenclature

CED: Corneal epithelial defect
PCED: Persistent corneal epithelial defect
RCE: Recurrent corneal erosion
PPV: Pars plana vitrectomy
NPDR: Nonproliferative diabetic retinopathy
PDR: Proliferative diabetic retinopathy
RD: Retinal detachment
HbA1c: Hemoglobin A1c
POD: Postoperative day
SB: Scleral buckle
PKP: Penetrating keratoplasty
BCL: Bandage contact lens
